# The impact of enteral immunonutrition on gut microbiota in colorectal cancer and gastric cancer patients in the preoperative period—preliminary results of randomized clinical trial

**DOI:** 10.3389/fimmu.2025.1606187

**Published:** 2025-08-07

**Authors:** Karolina Kaźmierczak-Siedlecka, Ewa Stachowska, Robert Kucharski, Piotr Wiśniewski, Paweł Ulasiński, Michał Stańczak, Wojciech Makarewicz, Karolina Skonieczna-Żydecka, Leszek Kalinowski

**Affiliations:** ^1^ Department of Medical Laboratory Diagnostics – Fahrenheit Biobank BBMRI.pl, Medical University of Gdansk, Gdańsk, Poland; ^2^ Department of Human Nutrition and Metabolomics, Pomeranian Medical University in Szczecin, Szczecin, Poland; ^3^ Neodentica Dentistry Center, Gdansk, Poland; ^4^ Unit of Surgery with Unit of Surgery with Unit of Oncological Surgery, Specialist Hospital in Koscierzyna, Kościerzyna, Poland; ^5^ Department of Surgical Oncology with the Subunit of Breast Cancer, Skin and Soft Tissue Surgery, Maritime Hospital PCK, Gdynia, Poland; ^6^ Oncological Surgery Department, Medical University of Gdansk, Gdynia, Poland; ^7^ 2nd Division of Radiology, Medical University of Gdansk, Gdańsk, Poland; ^8^ Department of Biochemical Research, Pomeranian Medical University in Szczecin, Szczecin, Poland; ^9^ BioTechMed Center, Department of Mechanics of Materials and Structures, Gdansk University of Technology, Gdańsk, Poland

**Keywords:** immunonutrition, gut microbiota, gastric cancer, colorectal cancer, surgery

## Abstract

**Introduction:**

Immunonutrition is a part of nutritional interventions in gastrointestinal cancer patients. It seems to be especially important in the preoperative period to reduce, among others, surgery-related complications. The relation between the immune system and gut microbiota has been previously analyzed. However, the influence of immunonutrients on the composition of gut microbiota is still unclear. Therefore, the aim of this study was to assess the impact of enteral immunonutrition on gut microbiota in gastric/colorectal cancer patients in the preoperative period.

**Patients and methods:**

This study included 14 patients (*n* = 9 colorectal cancer, *n* = 5 gastric cancer) allocated to receive immunonutrition or standard products by 7 days prior to the surgery. Randomization was performed using a random number generator. The stool samples were collected at day 0 and after 7 days of consuming the study products. Therefore, gut microbiota analysis was conducted at the beginning and after 7 days (follow-up). The analysis consists of alpha diversity analysis, taxonomic data processing, beta diversity analysis, and differential abundance analysis.

**Results:**

Statistical analysis did not indicate any significant differences (*p* > 0.05) between the two dietary groups for any of the alpha diversity indices. Microbial community compositions were largely similar between the immunodiet and standard nutridrink groups. Differential abundance analysis (DAA) using the Wilcoxon test identified several taxa with nominal *p*-values <0.05, suggesting potential differences in abundance between groups. However, none of these findings remained statistically significant. The taxa with nominal significance included *Bilophila*, *CAG-56*, *Clostridium sensu stricto 1*, *Coprobacter*, *Holdemania*, *Fusicatenibacter*, *Ruminococcus*, and [*Eubacterium*] *eligens* group.

**Conclusions:**

The analysis of gut microbiota in the context of immunonutrition is a new area in oncology. In the current study, despite some initial microbial alterations, it was not finally confirmed that immunonutrition has a beneficial effect on gut microbiota in gastric and colorectal cancer patients in the preoperative period. However, the small sample size is one of the study’s limitations.

## 1 Introduction

Immunonutrition is an integral part of complex nutritional support in cancer patients. Arginine, glutamine, omega-3 fatty acids, and nucleic acids are nutritional immunomodulatory substances which can be used in oncology (1–3). Patients with medium/high risk of malnutrition should receive immunonutrition orally/enterally in the perioperative period. This recommendation refers in particular to patients who will undergo surgery due to a tumor located in the upper part of the gastrointestinal tract (3–5). Immunonutrition provides multiple benefits. Among others, it enhances immune response, reduces the over-production of pro-inflammatory mediators, maintains CD4/CD8 lymphocyte balance, and contributes to a better clinical outcome (6, 7). It was meta-analytically demonstrated that such preoperative intervention decreases the prevalence of infectious complications, including wound infection, respiratory and urinary tract infections, and anastomotic leakage, by around 30% (8, 9). However, the data regarding the impact of immunonutrition on gut microbiome is still limited. Notably, the gut microbiome plays a significant role in the human body through maintaining intestinal barrier integrity (for instance, by producing short-chain fatty acids), reduction of secondary bile acids, increasing the level of anti-inflammatory cytokines, and carcinogen binding (10). Gut microbiome imbalance is observed in gastrointestinal cancers; however, the gut microenvironment imbalance depends on the type of cancer. In gastric cancer patients, an increased amount of *Helicobacter pylori*, *Lactobacillus*, *Escherichia*-*Shigella*, Nitrospirae, *Burkholderia fungorum*, and Lachnospiraceae is noted (11, 12). In the case of colorectal cancer patients, the abundance of *Escherichia coli*, *Enterococcus faecalis*, *Helicobacter hepaticus*, *Fusobacterium nucleatum*, *Porphyromonas gingivalis*, *Bacteroides fragilis*, *Peptostreptococcus anaerobius*, *Helicobacter pylori*, and *Streptococcus bovis* is observed (13). Additionally, in these patients, an imbalance of mycobiota (fungal part of microbiota) also occurs (10). Interestingly, in 2024, it was reported that overall insight into untargeted metabolomics in gastric and colorectal cancer patients showed that there is a link between the gut metabolome and both local and distal metastasis (14). The positive modulation of gut microbiota may restore microbial homeostasis, improve gut barrier integrity, and consequently contribute to the reduction of side effects of anti-cancer treatments (15).

Currently, as was mentioned above, there is a significant lack of data with regard the impact of immunonutrition in the perioperative period on gut microbiota composition (both bacteria and fungi). Additionally, in ClinicalTrials.gov system, there is only one trial registered (until February 5, 2025; keywords: cancer, immunonutrition, gut microbiota), except our trial (ClinicalTrials.gov system, identifier: NCT04980950), which assesses preoperative nutritional intervention and gut microbiota-related aspects. It actually concerns the case of head and neck cancer. Therefore, the aim of the present randomized trial is to assess the impact of enteral immunonutrition on gut microbiota in gastric/colorectal cancer patients in the preoperative period.

## 2 Patients and methods

The participants (*n* = 14, *n* = 9 colorectal cancer, and *n* = 5 gastric cancer) were recruited in the Unit of Surgery with the Unit of Oncological Surgery in Specialist Hospital in Koscierzyna, Koscierzyna Poland, Department of Surgical Oncology with the Subunit of Breast Cancer, Skin and Soft Tissue Surgery in Maritime Hospital PCK, Gdynia Poland, and Department of Surgical Oncology at Medical University of Gdansk, Poland. The patients were included to this study if they met all of the following inclusion criteria: age 18–80 years, presence of gastric/colorectal cancer, preparing for surgery due to tumor occurrence, and with written consent to take part in the study. The exclusion criteria included the following: age <18 years, presence of any type of cancer other than gastric/colorectal cancer, presence of any of inflammatory bowel diseases, pregnancy, or postpartum period. Randomization was performed using a random number generator. This study was conducted from 2021 to 2024. The study protocol has been approved by the Independent Bioethics Committee for Scientific Research at the Medical University of Gdansk (identifiers: NKBBN/129/2021, NKBBN/129-647,703/2021, NKBBN/129-281/2022, NKBBN/129-422/2022, NKBBN/129-126/2023, KB/129-378/2023).

### 2.1 Study design

#### 2.1.1 Study products

The patients who met all of the inclusion criteria and agreed to take part in this study were allocated to the intervention (I) or control group (N). Randomization with ratio 1:1 was performed. Therefore, in this 7-day, multi-center randomized study, 14 patients with gastric/colorectal cancer received enteral immunonutrition or consumed standard products at home. The post-allocation gut microbiota assessment was performed at days 0 and after 7 days of treatment in all of the participants. The surgery was planned at day 8. The patients in the intervention group (I, *n* = 9; colorectal cancer: *n* = 6, gastric cancer: *n* = 3) received one bottle of immunonutrition formula, i.e., Impact Oral Nestlé Health Science or Cubitan^®^ Nutricia, for 7 days. Oral products were given once a day in a dose of 237 mL of Impact Oral Nestlé Health Science and 200 mL of Cubitan^®^ Nutricia or Skin Repair. In the case of patients who could not swallow, immunonutrition (products: Impact Enteral Nestlé Health Science or Nutrison Advanced Cubison Nutricia) was planned to be given via artificial access to the gastrointestinal tract (gastrostomy, jejunostomy). The dose of enteral products should be calculated individually depending on the tolerance. It should be emphasized that the amount of active immunonutrient, i.e., arginine varies between 0.85 and 1.3 g/100 mL of enteral immunonutritional products—0.85 g/100 mL in Nutrison Advanced Cubison Nutricia and 1.3 g/100 mL in Impact Oral Nestlé Health Science. However, in this study, immunonutrition was taken only orally. In the control group (N, *n* = 5; colorectal cancer: *n* = 3, gastric cancer: *n* = 2), the participants consumed one bottle of the standard products Nutridrink^®^ Nutricia or Resource 2.0 Nestlé Health Science for 7 days. Both Nutridrink^®^ Nutricia and Resource 2.0 Nestlé Health Science were given in a dose of 200 mL. Similarly, in the case of patients who could not swallow, standard products, i.e., Nutrison Nutricia or Novasorce Gi Advance Nestlé Health Science, were planned to be given via artificial access to the gastrointestinal tract. However, it was not necessary.

#### 2.1.2 Stool samples

As was mentioned above, the stool samples (at least 4 g) were collected prior to the surgical treatment, i.e., (first sample) at day 0 before receiving immunonutrition/standard products and (second sample) after 7 days of consuming it. The stool samples were taken by the patients on their own, placed in a special tube (DNA/RNA Shield Fecal Collection Tube), and then provided to the researchers. Next, they were stored at -80°C in the Fahrenheit Biobank BBMRI.pl, Medical University of Gdansk, until the conduct of the gut microbiota analysis according to the well-established protocol at Sanprobi Sp. z o. o. Stool samples were sent to the Research and Development Center in Sanprobi for further processing. DNA was extracted from the stool samples using the MaqnifiQ Genomic DNA instant kit on an AutoPure Mini device. The protocol for fecal samples was combined with mechanical disruption using bead-beating homogenization. KAPA HiFi PCR Kit and Nextera XT Indexing kit were used. Purified DNA was used to prepare 16S metagenomic sequencing amplicon libraries (V3, V4) according to the Illumina protocol. NGS sequencing was performed in the Illumina system (MiSeq) using 300 bp paired-end reads.

#### 2.1.3 Participants’ timeline

The participants’ timeline is presented in **Table 1**. Each of the participants had two visits, i.e., at baseline and after 7 days of treatment. During the first visit, the participants received study products for 7 days.

**Table 1:** Schedule of enrollment, interventions, and assessments.

Time point	Study period					
	Enrollment	Allocation	Post-allocation			
	0	0	0	Day 7	Surgery	Analysis
Enrollment Eligibility screen	X					
Informed consent	X					
Allocation		X				
Interventions Immunonutrition given orally						
Standard products given orally						
Assessments Gut microbiota			X	X		X

## 3 Statistical analysis

The analysis was conducted using R version 4.4.2. These are the following analysis: alpha diversity analysis, taxonomic data processing, beta diversity analysis, and differential abundance analysis (DAA).

### 3.1 Alpha diversity analysis

The raw amplicon sequence variant (ASV) table was rarefied to a sequencing depth of 93,144 reads per sample. Rarefaction was performed using the rtk package (version 0.2.6.1). The rarefied data was used to calculate alpha diversity indices: richness, evenness, Simpson’s diversity index, and Shannon diversity index. To assess differences in alpha diversity indices between dietary groups, Wilcoxon rank-sum test was performed (Mann–Whitney *U*-test). Boxplots were generated for each diversity metric (richness, evenness, Simpson, and Shannon indices) using the ggplot2 package.

### 3.2 Taxonomic data processing

The proportion of unassigned and uncultured taxa was computed before removing these taxa from the dataset. The mean proportion of unassigned/uncultured taxa was 21.81%. The filtered dataset was then rarefied to a depth of 68,636 reads per sample, following the same rarefaction process as described earlier. Prevalence filtering was applied to retain features present in at least 20% of the samples, removing low-prevalence taxa. A total of 36 features were filtered out.

### 3.3 Beta diversity analysis

Beta diversity was estimated using Bray–Curtis dissimilarity, calculated using the vegdist() function from the vegan package (version 2.6-10). A principal coordinate analysis (PCoA) was performed, and the first two principal coordinate axes (PCoA1 and PCoA2) were extracted. To assess differences in community composition between dietary groups, PERMANOVA was conducted using the adonis2() function with 999,999 permutations. The PCoA scores were visualized with ggplot2, with ellipses representing variance within dietary groups.

### 3.4 Differential abundance analysis

The rarefied taxonomic dataset was transformed, and relative abundances of taxa were computed. Differential abundance testing was performed using Wilcoxon rank-sum tests, iterating over each genus to compare relative abundances between dietary groups. Bonferroni correction was applied to adjust for multiple comparisons (0.05/147). A Manhattan plot was generated to visualize *p*-values using ggplot2, with significance thresholds denoted by horizontal dotted lines.

## 4 Results

This study included 14 patients (*n* = 9 colorectal cancer and *n* = 5 gastric cancer). The analysis is based on two groups, i.e., patients who received immunonutrition (I) and patients consuming standard products (N). The participants from both groups received products within 7 days in the preoperative period.

**Figure 1:**
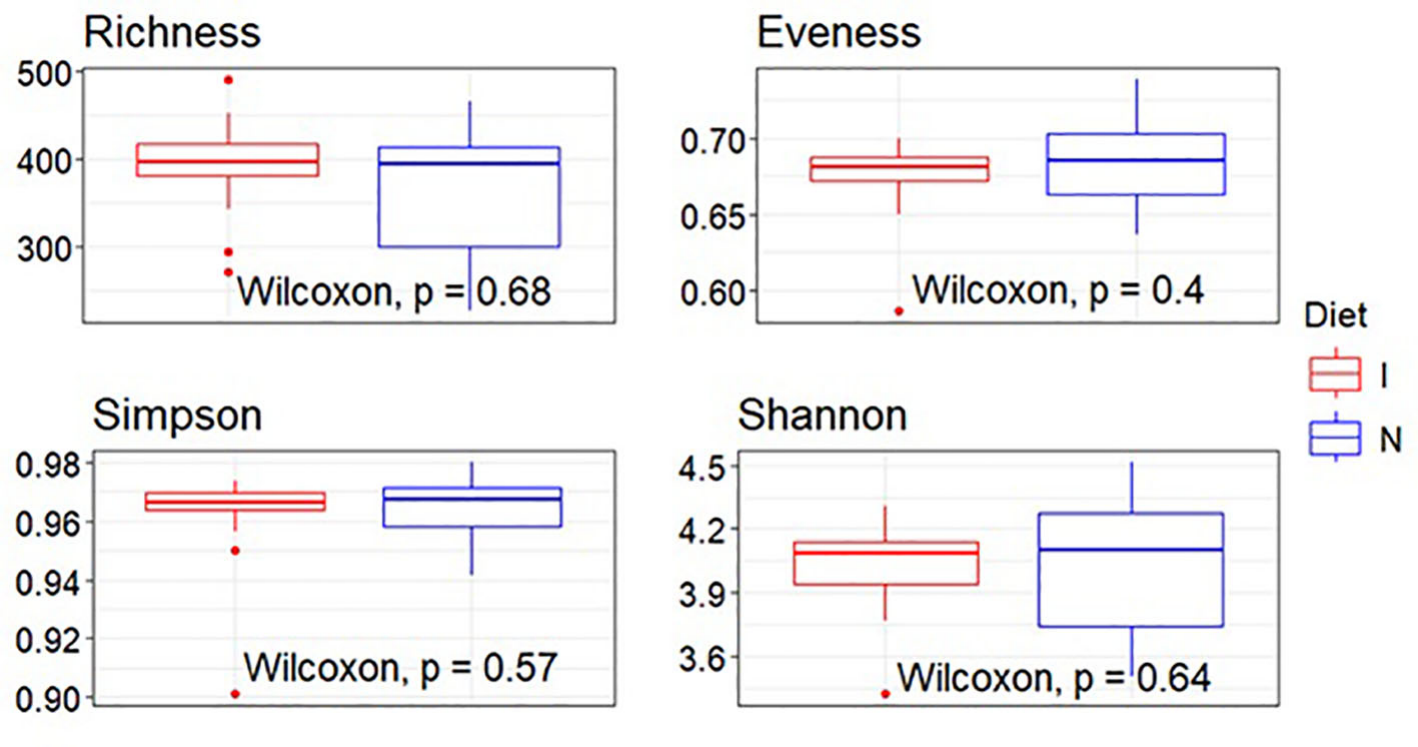
Boxplot visualization of alpha diversity indices (richness, evenness, Simpson, and Shannon). Each boxplot represents the interquartile range (IQR), with the median indicated by a horizontal line within the box. Whiskers extend to the minimum and maximum values within 1.5 times the IQR, while outliers are shown as individual points. Wilcoxon rank-sum tests were performed to assess statistical significance, with *p*-values annotated directly on the plots. Colors were assigned manually to differentiate dietary groups: I, immunodiet—red; N, standard nutridrink—blue.

The summary statistics for alpha diversity indices grouped by diet indicate similar distributions across the two groups. The richness index, representing the number of unique taxa, showed a mean of 392.51 (SD: 56.14) for the immunodiet group (I) and 366.64 (SD: 84.73) for the standard nutridrink group (N). Evenness, which measures the uniformity of species distribution, had mean values of 0.674 (SD: 0.041) for group I and 0.686 (SD: 0.042) for group N. Simpson’s index, reflecting species dominance, remained high for both groups (mean 0.969 for I and 0.971 for N), suggesting stable community structures. Shannon’s index, incorporating both richness and evenness, showed comparable means (4.02 for I and 4.04 for N). There was no significant differences between the two dietary groups for any of the alpha diversity indices as visualized in the boxplot representation (**Figure 1**).

**Figure 2:**
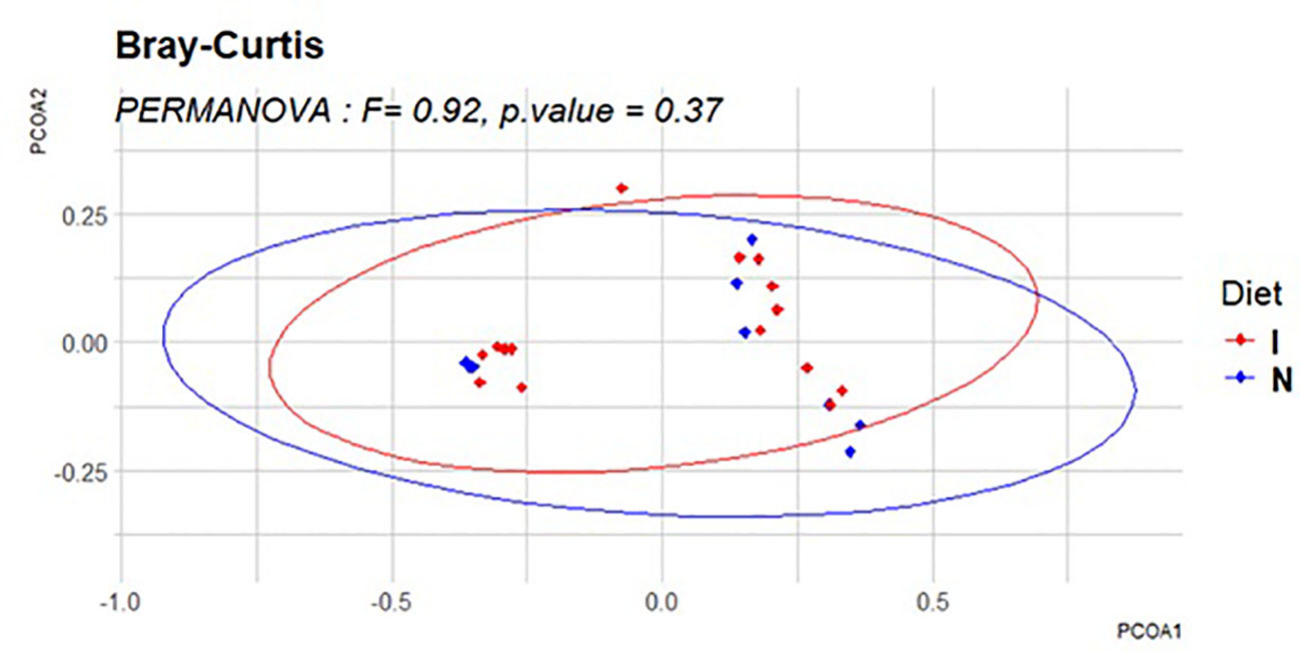
Principal coordinate analysis (PCoA) of beta diversity. The PCoA plot visually represents beta diversity differences between dietary groups using Bray–Curtis dissimilarity. Each point corresponds to a sample, and its position in the plot reflects the microbial community composition. Ellipses indicate the variance within each dietary group.

**Figure 3:**
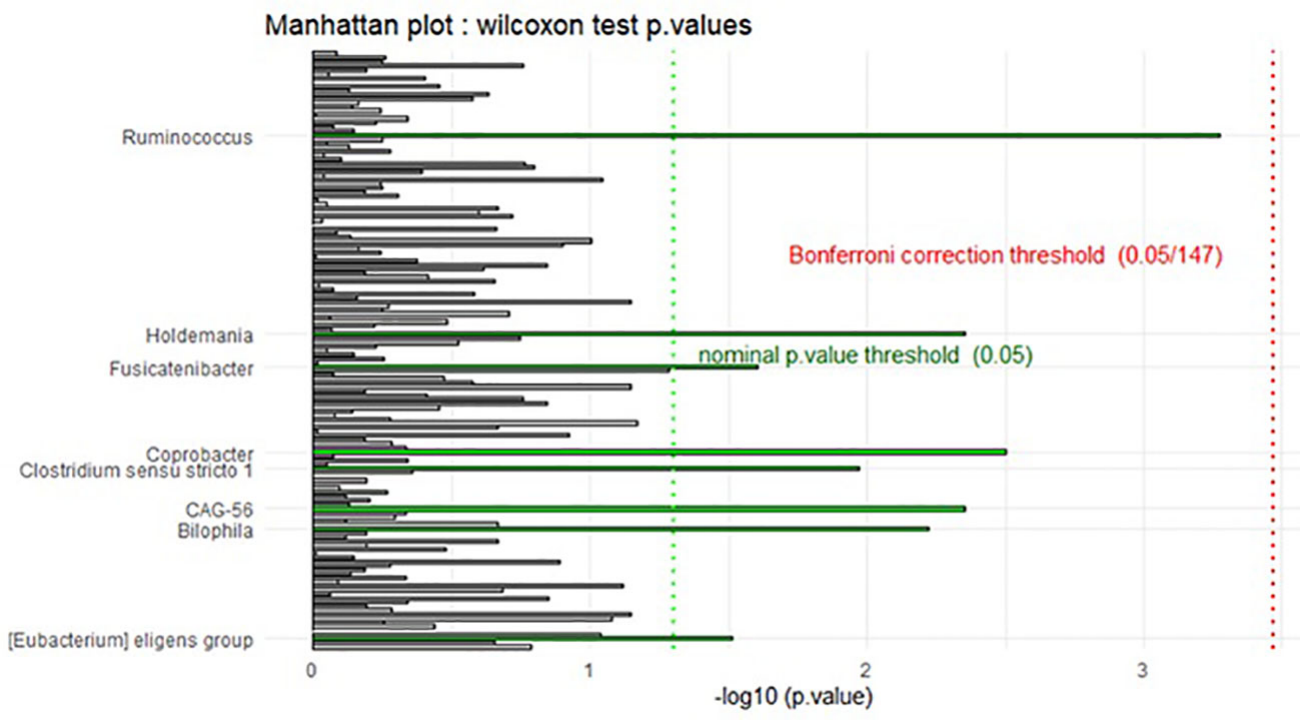
Manhattan plot of differential abundance analysis (DAA). The Manhattan plot provides a visual representation of the differential abundance analysis results. Each bar represents a genus, with the x-axis displaying the -log10(p-value), indicating the statistical significance of differences in abundance between dietary groups. The dotted vertical lines denote significance thresholds: the green line represents the nominal *p*-value threshold (0.05), while the red line indicates the Bonferroni correction threshold (0.05/147). Bars are colored according to significance classification, highlighting taxa that exhibit statistically significant differences.

The PERMANOVA analysis indicated that there were no statistically significant differences in beta diversity between the dietary groups (*F* = 0.92, *p* = 0.37). This suggests that microbial community compositions were largely similar between the immunodiet and standard nutridrink groups. The PCoA plot further supports this finding, as the points corresponding to both groups exhibit a substantial overlap, indicating that the dietary interventions did not induce distinct clustering of microbial communities (**Figures 2**, **3**).

Differential abundance analysis (DAA) using the Wilcoxon test identified several taxa with nominal *p*-values <0.05, suggesting potential differences in abundance between groups. However, after applying the Bonferroni correction for multiple comparisons, none of these findings remained statistically significant. The taxa with nominal significance included *Bilophila*, *CAG-56*, *Clostridium sensu stricto 1*, *Coprobacter*, *Holdemania*, *Fusicatenibacter*, *Ruminococcus*, and [*Eubacterium*] *eligens* group.

## 5 Discussion

In the current study, the impact of immunonutrition given orally on the composition of gut microbiota in gastric/colorectal cancer patients in the preoperative period was analyzed. Enteral immunonutrition may provide many benefits to gastrointestinal cancer patients, such as boosting the immune system, promoting tissue repair (especially arginine) as well as improving the nutritional status (16). Arginine, which is known as a nutritional immunomodulatory substance, takes part in wound healing process. Thus, its administration in the perioperative period may be significant (1). It should be mentioned that arginine is a non-essential amino acid; however, it can be conditionally essential in cases of cancer/trauma. In systematic review and meta-analysis, it was noted that perioperative immunonutrition in patients with gastrointestinal cancers reduces the complications after surgery and the length of hospital stay (17). In another systematic review and meta-analysis (seven studies, *n* = 583), Cheng et al. (18) have shown that enteral immunonutrition modulates inflammatory reaction and reduces postoperative complications in gastric cancer patients undergoing total gastrectomy. In colorectal cancer patients undergoing surgery, enteral immunonutrition also shortened the length of hospital stay (pooled mean difference, 2.53; 95% CI, 1.29–3.41) and reduced infectious complications (pooled odd risk, 0.33; 95% CI, 0.21–0.53) (9). Immunonutrition stimulates the immune system. In a randomized, double-blind, controlled trial, Li et al. (19) proved that early postoperative enteral immunonutrition enhances the function of the immune system in gastric cancer patients. Similar results were obtained in another study in which it was noted that early enteral immunonutrition promotes the immune function in gastric cancer patients (20). The level of CD4+/CD8+ was significantly higher in the group receiving immunonutrition than in control subjects (*P* < 0.05) (20).

The gut microbiome affects the anti-cancer treatment efficacy. Among others, it has an impact on the effectiveness of the surgical treatment. Anastomotic leak is a complication in colorectal surgery with multifactorial etiology including the interaction between host genetics, inflammation, and gut microbiome (21–24). Bacteria, such as *Enterococcus faecalis* and *Pseudomonas aeruginosa*, participate in the development of an anastomotic leak (25, 26). Low gut microbial diversity and the abundance of mucin-degrading bacteria (*Lachnospiraceae* and *Bacteroidaceae* families) also contribute to anastomotic leak occurrence (27). Moreover, damage of tight junction and increased intestinal permeability in gastrointestinal cancer patients are observed (28). As was mentioned above, previously published studies concentrated mainly on the influence of immunonutrition on surgical-related complications. However, the data regarding its impact on gut microbiota in cancer patients is limited. Therefore, in the current study, we focused on immunonutrition and gut microbiome in gastric and colorectal cancer patients. Nevertheless, the summary statistics for alpha diversity indices grouped by diet indicate similar distributions across the two groups, i.e., I and N. Therefore, statistical analysis did not indicate any significant differences (*p* > 0.05) between the two dietary groups for any of the alpha diversity indices. Similarly, this was also true in the case of beta diversity. However, DAA using the Wilcoxon test identified several taxa with nominal *p*-values below 0.05, suggesting potential differences in abundance between groups. The taxa with nominal significance included *Bilophila*, *CAG-56*, *Clostridium sensu stricto 1*, *Coprobacter*, *Holdemania*, *Fusicatenibacter*, *Ruminococcus*, and [*Eubacterium*] *eligens* group. In the further analysis, it was noted that none of these findings remained statistically significant. It should be emphasized that some of the listed microbes act with pro-inflammatory properties—for instance, *Bilophila* increases the risk of intestinal permeability. *Ruminococcus gnavus* presents pro-inflammatory activity and promotes the development of gut dysbiosis (29, 30). Nevertheless, some strains of *Ruminococcus* are known as a SCFA producers; thus, they can exert beneficial effects (31).

## 6 Conclusions

The analysis of gut microbiota in the context of immunonutrition is a new area in oncology. Restoration of the gut microbiota balance is strongly needed in gastrointestinal cancer patients. Therefore, modern methods of gut microbiota modulation should be found. Notably, immunonutrients, immunometabolism-related aspects, mucosal immune system, and its interactions with gut microbiota seem to be crucial, and it is a new perspective for cancer patients. In the current study, microbial community compositions were largely similar between the immunodiet and standard nutridrink groups. However, differential abundance analysis identified several taxa with nominal *p*-values below 0.05, suggesting potential differences in abundance between the studied groups. Nevertheless, despite some initial microbial alterations, it was not finally confirmed that immunonutrition has a beneficial effect on gut microbiota in gastric and colorectal cancer patients in the preoperative period.

## 7 Limitations and future directions

There are possible limitations in the design/conduct of the study, such as the following:

• small sample size (*n* = 14), limiting the statistical power

• short duration of intervention (7 days)

At the beginning, the post-allocation gut microbiota assessment was planned at day 0 and after 7 and 10 days, respectively, of treatment in all participants. The surgery was planned at day 8. Therefore, the study products’ administration was planned to be interrupted after the first 7 days, and then it was planned to be continued 1 day after the surgical procedure. It could (1) provide the impact of surgical treatment on gut microbiota in a short time and (2) show the changes of microbiota caused by the consumption of immunonutrients for a longer time (10 days instead of 7 days).

• Uncertainty regarding compliance with supplementation.

Next, the patients received the study products at home. Therefore, the researchers cannot be completely sure if they consumed the products. However, the participants were asked about compliance at follow-up visits.

The future direction is with regard the assessment of the impact of immunonutrition on microbiome-derived metabolites in cancer patients. In the current study, it was not finally confirmed that immunonutrition has a beneficial influence on overall gut microbiota. However, the alterations could be observed in the level and proportion of gut-microbiome-related metabolites (for instance, short-chain fatty acids). The aspects of untargeted metabolomics also seem to be crucial in this context.

## Data Availability

The data have been deposited and the accession for raw data is below: Accession to cite for these SRA data: PRJNA1289017 https://www.ncbi.nlm.nih.gov/sra/?term=PRJNA1289017.
